# Semantic segmentation of methane plumes with hyperspectral machine learning models

**DOI:** 10.1038/s41598-023-44918-6

**Published:** 2023-11-17

**Authors:** Vít Růžička, Gonzalo Mateo-Garcia, Luis Gómez-Chova, Anna Vaughan, Luis Guanter, Andrew Markham

**Affiliations:** 1 University of Oxford, Oxford, UK; 2 Trillium Technologies, London, UK; 3 University of Valencia, Valencia, Spain; 4 University of Cambridge, Cambridge, UK; 5 Polytechnic University of Valencia, Valencia, Spain; 6 Environmental Defense Fund, Amsterdam, Netherlands

**Keywords:** Environmental sciences, Natural hazards, Atmospheric science, Climate change, Computer science, Scientific data, Software

## Abstract

Methane is the second most important greenhouse gas contributor to climate change; at the same time its reduction has been denoted as one of the fastest pathways to preventing temperature growth due to its short atmospheric lifetime. In particular, the mitigation of active point-sources associated with the fossil fuel industry has a strong and cost-effective mitigation potential. Detection of methane plumes in remote sensing data is possible, but the existing approaches exhibit high false positive rates and need manual intervention. Machine learning research in this area is limited due to the lack of large real-world annotated datasets. In this work, we are publicly releasing a machine learning ready dataset with manually refined annotation of methane plumes. We present labelled hyperspectral data from the AVIRIS-NG sensor and provide simulated multispectral WorldView-3 views of the same data to allow for model benchmarking across hyperspectral and multispectral sensors. We propose sensor agnostic machine learning architectures, using classical methane enhancement products as input features. Our HyperSTARCOP model outperforms strong matched filter baseline by over 25% in F1 score, while reducing its false positive rate per classified tile by over 41.83%. Additionally, we demonstrate zero-shot generalisation of our trained model on data from the EMIT hyperspectral instrument, despite the differences in the spectral and spatial resolution between the two sensors: in an annotated subset of EMIT images HyperSTARCOP achieves a 40% gain in F1 score over the baseline.

## Introduction

Methane leak detection from anthropogenic sources has seen increasing attention, as it is regarded as one of the most viable targets for preventing catastrophic scenarios in temperature increase due to climate change related effects^1^. Given methane’s short atmospheric lifetime, its removal from the atmosphere would have a very rapid effect in reducing global warming over the next decades. Large leaks, the so-called super-emitters, have been shown to contribute disproportionately to the concentration of methane in the atmosphere: Lavaux et al.^2^ recently showed that 12% of all oil and gas (O &G) methane emissions are episodic ultra-emission events that in many cases are caused by equipment failures in oil rigs, pipelines or well pads. Additionally, those emissions are highly underestimated: Alvarez et al.^3^ reported that O &G supply chain emissions in 2015 were 60% higher than bottom up estimates from the United States Environmental Protection Agency, and Zhang et al.^4^ reported that observed emissions using satellite data are two times higher than bottom-up inventories in the Permian basin. This is due to the fact that bottom-up inventories often underestimate emissions, which can be improved with the use of satellite-based information.

Using different multispectral and hyperspectral satellite instruments several works^5–9^ have proposed methods for detection and identification of point sources of medium to large methane emissions (>100kg/h). However these methods still require a significant amount of manual intervention: for hyperspectral instruments, methods based on a matched filter, such as mag1c^10^, produce reliable enhancements, however, they are still prone to high false detection rates. Meanwhile, methods for multispectral data have not been automated and existing approaches^6,8^ require manual inspection by human experts looking at pre-computed spectral ratio products. Furthermore, there is no standard dataset for the task of methane plume detection; existing works^5–9^ report detection limits anecdotally and do not allow for an easy comparison between methods and sensors. Additionally, research in machine learning models aimed at processing hyperspectral data is limited, uses very small datasets, and usually focuses on the task of land cover classification.

With the arrival of recent satellite missions, such as PRISMA^7,11^, EnMAP^12^ and the NASA’s Earth Surface Mineral Dust Source Investigation (EMIT)^13^ there is a need for a reliable automated method with low false detection rate capable of automatically detecting methane plume leaks that would also enable methane plume attribution. In this context, research on transferability of the knowledge from data collected from one sensor, ideally with reliable annotations, to other novel sensors would be highly useful^14^.

The aim of this paper therefore is to address these problems and to foster artificial intelligence (AI) research in this area. For this, we improve and extend the annotation of the dataset of AVIRIS hyperspectral images from the Permian basin aerial campaign^15^. We release the extended annotation with this dataset in a machine learning ready format, that can serve as a testbed for further research in methane plume detection and in general for processing hyperspectral data with machine learning models. For a more in-depth analysis of the methane events present in the dataset, we refer the reader to^15^. In our dataset, we include 1878 images of high quality with verified plume events, which are matched with an equal number of background class samples with no observed emissions.

**Figure 1:**
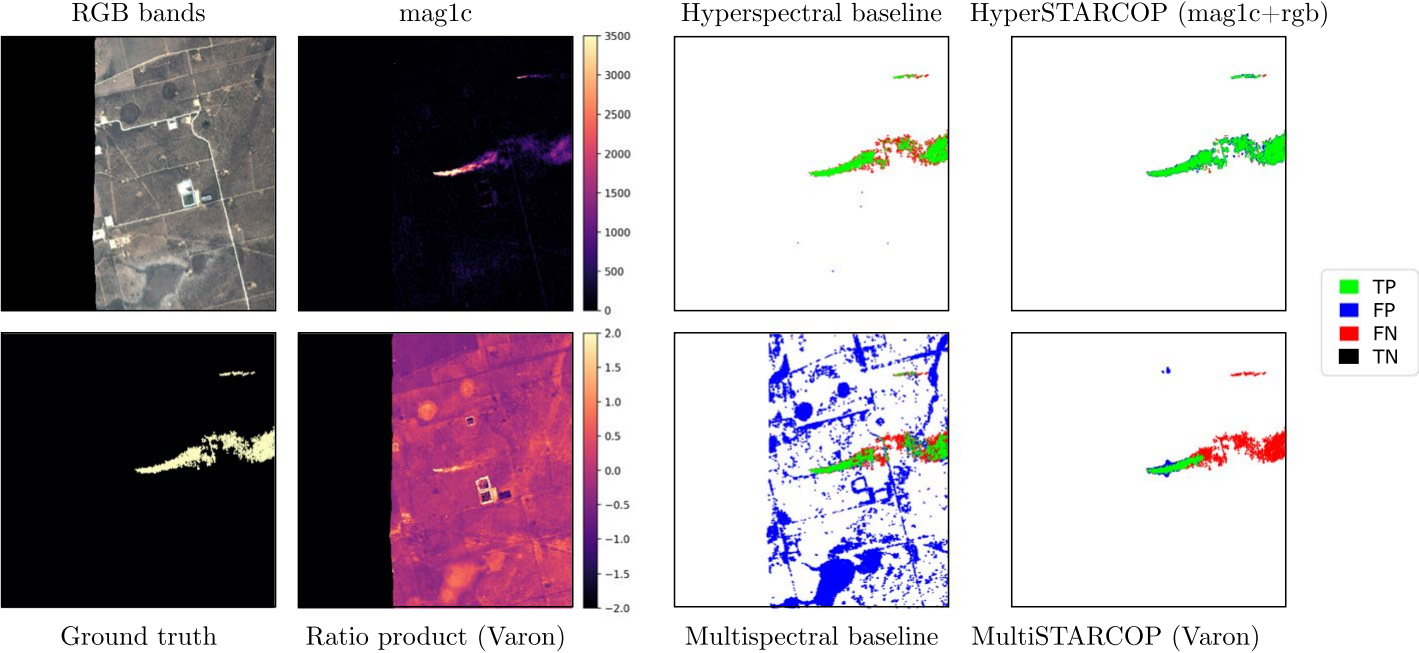
Predictions on an example plume event from the AVIRIS data. On the left, we show some of the inputs used by our models and by the baselines and, on the right, we show the comparison of the predictions with the ground truth labels. It can be seen, that the major issue with the existing baselines is the high false positive ratio.

Furthermore, we propose small and efficient machine learning models based on the U-Net architecture^16^ that use the established representations for both hyperspectral and multispectral data. Our model is also lightweight, with only 6.6M parameters in total. For hyperspectral data, we show that, using our HyperSTARCOP model, we can significantly reduce the false detection rate of mag1c^10^, while maintaining high semantic segmentation performance. For simulated WorldView-3 multispectral data, we propose a MultiSTARCOP model that enables automatic methane detection using existing pre-computed band ratios^6,8^. Figure 1 shows an example prediction of the proposed models in comparison with the existing baselines.

Finally, we demonstrate sensor agnosticism of our proposed method, by showing that the models trained on the AVIRIS dataset work as zero-shot detectors on images from a different hyperspectral sensor such as EMIT. These two sensors have vastly different properties, resolutions, deployment and also scope: the AVIRIS training data is from the Permian Basin region in the US and collected aerially, while space based sensor EMIT acquires imagery on all arid and semi-arid regions where most oil and gas fields are located. We note, that our approach would likely work with data from other hyperspectral sensors as well.

To summarise, the benefit of having an automated methane detection system is clear in the context of the ever-increasing size of Remote Sensing data and with the expected increased data collection cadence of the upcoming hyperspectral satellite missions. Such an automated system would ease the work of experts in this field, as it could sift through the vast amounts of data and propose locations of interest for manual confirmation and release through relevant agencies. Another interesting direction would be the deployment of our system for fully autonomous detection of methane plumes on-board of satellites to allow for increased autonomy of satellite constellations. Suspected detections could trigger automated scheduling of follow-up observations of the same area, potentially increasing the capture of scientifically interesting data. Finally, using the methods presented by^17,18^, it is possible to quantify methane emissions using the methane enhancement products and publicly available wind information. Using our system, one can effectively clean up typical confounders from these products, and arrive at better estimates of plume quantities.

## Background

### Methane signature and enhancement methods

**Figure 2:**
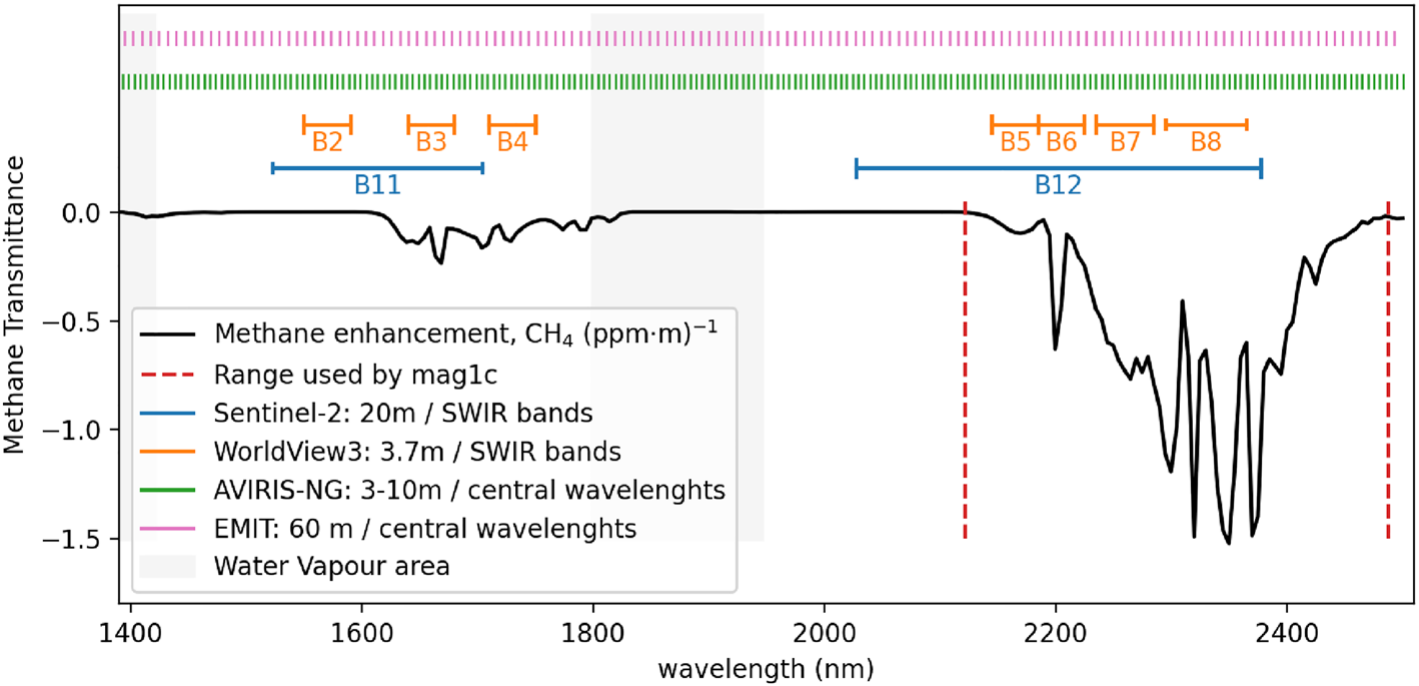
Illustration of the presence of the methane signal (shown through the methane transmittance) in comparison with the bands available in commonly used satellites. For clarity the hyperspectral sensors of AVIRIS-NG and EMIT, show the central wavelengths, while for the multispectral instruments of Sentinel-2 and the WorldView-3, we show the whole band ranges in the short-wave infrared (SWIR) region. We also highlight the region that corresponds to water vapour absorption, which is typically excluded from the data analysis.

The methane signal in the near-infrared part of the electromagnetic spectrum is visible mainly in two spectral ranges: between the wavelengths of 1600 and 1850 nm, and between 2100 and 2500 nm. This is relevant for the choice of the instrument, as we likely want to capture at least one of these regions, and usually also a region outside of these ranges, to get information about the background signal. There are several satellites capable of observing methane concentrations with spectral bands on these ranges with various spatial resolutions^9^. On the one hand, hyperspectral imagers such as AVIRIS or EMIT cover both methane ranges at narrow spectral resolution (5–8 nm). On the other hand, multispectral satellites such as WorldView-3 or Sentinel-2 have broad bands overlapping those spectral ranges. Controlled methane release experiments have validated the retrieval methods on both airborne and satellite instruments^19–21^. Figure 2 shows the intersection of the expected signature of the methane signal with different types of satellites referenced in this paper.

Uniquely, as compared with other semantic segmentation tasks conducted with Remote Sensing data, the methane plumes are not visible in any one isolated band. A vast array of different enhancement methods are therefore used to highlight the plume inside the data. In this work, we explore these products as inputs of the proposed models. For multispectral instruments (MSI) we explore the ratio products^6,8^, while for hyperspectral instruments (HSI) we use the improved matched filter approach^10^.

We note that, in practice, most of the mentioned approaches remain manual. A visual inspection by human experts is needed, often with a requirement of parameter tweaking given different locations. Most of these methods produce many false positive detections. Furthermore, it is not obvious how to use these methods in an automated manner out of the box. In this paper, we use these classical approaches to extract relevant features of high dimensional input data, and we use thresholding methods to explore the feasibility of their direct implementation as baselines.

### Machine Learning for hyperspectral data processing

Research in machine learning for hyperspectral data processing is limited mainly because there is a lack of relevant, large and annotated datasets with hyperspectral data, that have high spatial resolution and diverse geographical distribution across the world^22^. Existing benchmarks such as Indian Pines^23^ or University of Pavia are based on one or very few small image acquisitions. As noted in several overview papers^24–26^, this works particularly badly for the hyperspectral scenario, as the high dimensionality of the data, in combination with the low number of samples, introduces severe problems connected with the curse of dimensionality for the trained models. This is sometimes addressed by using simulated data, or private datasets^26^. To make matters worse, the typically taken approach is to divide this already small data into training and test sets, and the reported scores tend to be heavily overfitted, giving very large accuracy numbers almost regardless of the used method^25^. Finally, most of the existing hyperspectral datasets focus on per pixel classification of land cover classes while other problems where the hyperspectral signature would be more interesting are not available.

### Machine learning for methane detection

Research in machine learning models to be used with remote sensing data for methane detection has been limited. A large amount of the work also remains manual. For example, the work of^27^ has used machine learning models to detect methane plumes in the extremely coarse and low resolution data of the TROPOMI sensor, and has used these automated detections for a manual search in higher resolution data. Similarly, there have been works on researching methane enhancement products for multispectral data – using Sentinel-2 and Landsat data^5,6,28^ and using the WorldView-3 data^8^. However, these works still require follow-up manual intervention, and it is difficult to estimate the performance of each of these enhancement methods in novel untested locations and when compared against each other. There is no benchmark dataset available in this domain that would permit fair comparison across different modalities of the data.

There have been very recent works^29–31^ applying deep learning to hyperspectral data with simulated methane plumes. The workshop paper of^30^ frames the detection of methane plumes as semantic segmentation and uses the matched filter product generated using data from the on-demand satellite PRISMA. They create a synthetic dataset by combining the plume maps from 1000 Sentinel-2 images^28^ with real, matched filter backgrounds from 150 plume-free PRISMA images. The work of^29^ instead focuses on the regression task of estimating the emission rate from methane enhancement products. They generate artificial plume shapes using the Large Eddy Simulation (LES)^32^ and mix these with the background noise of matched filter outputs from the AVIRIS data. Finally, the preliminary work of^31^ combines the tasks of semantic segmentation with regression, by sequentially training several models to first segment and later quantify the methane emissions from the PRISMA satellite images. Similarly, as in the other instances, the annotation is made by methane plume simulations using the LES and mixing the generated signal back into the hyperspectral data. Unfortunately none of these works provide the datasets and data products and code and the trained models are also not open-sourced.

In our view, the contributions of our work compared to these recent works are that (i) our paper considers a dataset made of real-world methane plumes instead of synthetic simulated plumes, (ii) our curated dataset is larger (from 300 different AVIRIS acquisitions) although it is limited to the Permian basin area (iii) we train and compare models from multispectral and hyperspectral views of the data iv) we show zero-shot transferability of the proposed hyperspectral model to other sensor and v) we open-source the dataset with the curated labels, the codebase and the trained models.

## Data

**Figure 3:**
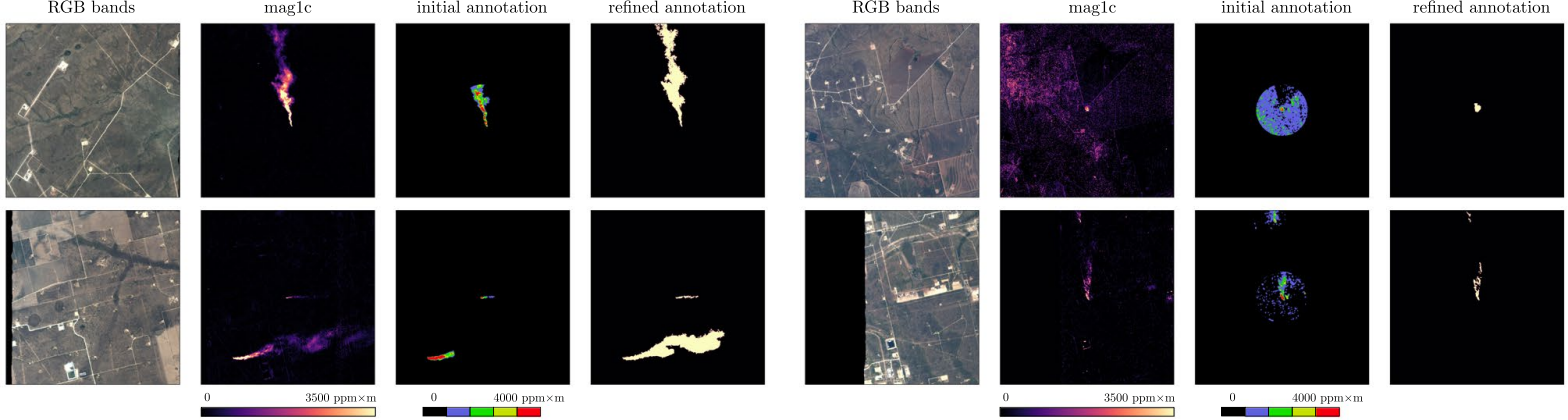
Showing the limitations of the available annotation in the original labels released by^15^, with a circular mask cut out around the center of each plume. We manually refine and extend these plumes using the mag1c product as guidance.

One of the purposes of our study is to compare the capacity to segment methane plumes of different models on different data modalities but under the same conditions (i.e. using the same dataset). For this, we constructed a balanced ML-ready dataset with different image instances for each element in the dataset. Those instances are manually annotated plume masks, $\text {CH}_4$ retrievals using the standard enhancement products, hyperspectral images and multispectral images simulated using the AVIRIS-NG hyperspectral data. In order to test the generalization capabilities of our hyperspectral model, we additionally collected a set of images from the EMIT hyperspectral sensor with verified emissions that we also manually labeled.

### AVIRIS machine learning ready dataset

The data of the Permian basin airborne campaign conducted for the study of Cusworth et al.^15^ was selected to create our dataset. This campaign was conducted from September to November of 2019. 3068 individual methane plumes were found in 564 images retrieved on 31 different days using two hyperspectral airborne instruments: the Next-Generation Airborne Visible/Infrared Imaging Spectrometer (AVIRIS-NG) and the Global Airborne Observatory (GAO) instrument^33^. Images retrieved by those instruments have an spectral resolution of 5–10 nm and a spatial resolution of 3–10 m. The location, time of acquisition and rough plume segmentation mask of those plumes is available at Zenodo^34^. Since data from the GAO sensor is not public, we used only the plumes retrieved by AVIRIS-NG. The AVIRIS-NG flight lines of this campaign correspond to 300 level 1B hyperspectral images with an approximate size of 6TB. For each of these images, we derive: the $\text {CH}_4$ enhancement using the mag1c matched filter model proposed in^10^ and the simulation of the bands of WorldView-3 using the corresponding spectral response function. We describe each of these in detail in the following subsections. The final dataset size, which provides the relevant and used bands and products, is 60 GB.

We sampled chips of size 512$\times$512 px from the available AVIRIS-NG images to create the STARCOP dataset. In order to obtain a balanced dataset we selected all tiles containing plumes as positive examples (*plume*) and sample the same amount of tiles from the pool of locations without plumes to be negatives samples (*no-plume*). For the negative samples, half of them are randomly selected whereas the other half is chosen using the mag1c^10^ product as locations with high amount of confounders, i.e. locations where mag1c output is high but there are not plumes.

Plume masks provided in original data^15^ are in a colour mapped RGB PNG format covering a 151$\times$151 pixel area. Our first tests with these labels found several inconsistencies such as build up areas near the plume labelled as plume or labels covering only a circular area around the plume source – these are shown in Fig. 3. Also, our chips are larger (512$\times$512) which was a problem since the original labels only covered 151$\times$151 pixels. Since data quality is of paramount importance for ML models, we manually extended and curated the labels using the IRIS tool (Intelligently Reinforced Image Segmentation) graphical user interface^35^, which was previously used for similar tasks in^36^. We mainly used the brush tool to remove label errors and extend the plume to capture the tails. Additionally, we manually inspected all *no-plume* locations to make sure no plume is present in these chips since we found a couple of large plumes not reported in the original dataset^34^.

**Figure 4:**
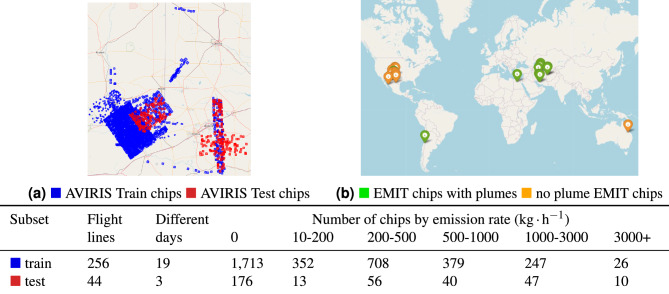
ML-ready dataset produced in this study. **(a)** Location of the chips of size 512$\times$512 pixels used for training and testing the proposed system in the Permian basin. **(b)** Location of the EMIT granules used for evaluation of zero-shot detection. In the bottom table: Statistics of the number of flight lines, days and amount of chips stratified by emission rate.

In order to split the chips for training and testing, we manually selected chips coming from acquisitions of three days (18th, 21st and 25th of October) to avoid temporal overlap. We chose these days as they are clustered towards the end of the campaign and they have a balanced amount of plumes of different strengths. Image acquisitions from other days are used for training the models. Figure 4a shows the spatial location of AVIRIS training and testing tiles, and the statistics of training and testing chips stratified by the emission rate. For evaluation, we label the data in the test dataset with broad categorical labels: plumes with emission rate lower than 1000 kg/h are labelled as “strong”, while the rest is labelled as “weak”.

In order to simulate retrievals of multispectral sensors from AVIRIS-NG images, we convolve the hyperspectral bands with the spectral response function (SRF) of the sensors we seek to simulate. We further converted the radiance values to top of atmosphere (TOA) reflectance using the date of acquisition of the image, the center location and the solar irradiance. For WorldView-3 the solar irradiance on each of the bands is obtained by convolving the SRF of the sensor with the Thuillier solar spectrum^37^. We have also experimented with the Sentinel-2 satellite as the target for the simulated data, however, even the largest of methane plumes in the Permian Basin area were not visible in the Sentinel-2 band ratio products^6,18^.

We note that with the released dataset we provide the community a tool to simulate synthetic data for any target sensor working in this spectral range, given the knowledge of the properties of the captured bands such as the spectral response function. Similar ratio products would be used. We warn that in some cases this likely wouldn’t work with this particular set of plumes due to the low methane concentrations (as we saw in the case of Sentinel-2). We also note that our dataset can serve as a source of information about the shapes and intensities of methane plumes, which could be used for simulation of new labelled datasets^29–31^.

We use several data augmentation techniques on the samples in our training dataset. We apply random rotations and extract 128$\times$128 px tiles with an overlap of 64 px from the original 512$\times$512 px scenes. With this approach our final training dataset holds 167,825 images, each with all the required intermediate feature products. Our test dataset is kept as 512$\times$512 px scenes.

In summary, we release the final dataset with labelled methane plumes from the AVIRIS-NG sensor alongside the refined labels, simulated multispectral products and added enhancement products (these are described in more details in the Methodology section). The dataset contains in total 1878 plume events, which are split between train and test datasets and samples with no plumes (from random locations and from areas with known confounders). Our dataset is available at: https://doi.org/10.5281/zenodo.7863343^38^.

### EMIT dataset for estimation of generalisation ability across hyperspectral sensors

In order to explore the generalisation ability of our proposed models, we have tested the hyperspectral models trained on the AVIRIS-NG data on a small dataset from another hyperspectral sensor, EMIT. Given the inputs used in our proposed model, we are able to extract similar image patches from another sensor, despite it having different spatial and spectral resolutions.

Most importantly, the geographic locations where the data was collected differ – AVIRIS-NG was collected locally in the Permian Basin area of USA, while EMIT is global, covering arid regions around the world. Additionally, the AVIRIS-NG sensor is an aerial based mission, while the EMIT sensor is deployed on board of the International Space Station (ISS). This does have an influence on the amount of atmospheric disturbances affecting the data. Both the ground and the spectral resolution between the two sensors also differs as can be seen on Fig. 2. We are able to circumvent the dependency on one particular sensor, by using a matched filter product as one of the inputs to our models.

We have annotated a small subset of the data from the EMIT sensor, which serves as an evaluation dataset. The selection was based on the initially released labels through the EMIT Open Data Portal. In total, we have selected and manually annotated 11 locations with known plumes and 9 locations without any reported plumes – as shown on Fig. 4b. We use the released L1 level of processing of the EMIT data without applying orthorectification, this is in a conscious attempt to simulate near raw data, which would be available on-board of the sensor.

## Methodology

As has been discussed in the previous section, we have compiled a dataset of hyperspectral images, which allows us to simulate multispectral views of the same data. This gives us an option to design machine learning models operating on both types of data and to compare their performance. In this section we describe these models, the proposed feature extraction of inputs depending on the data modality (multi or hyperspectral) as well as the baselines that are compared against.

### Feature extraction from multispectral data

Multispectral data is expected to give lower detection capabilities of methane plume detection, namely due to the lower intersection between available bands and the methane spectral absorption signature as seen on Fig. 2. This is typically addressed by comparing a single band that falls within the methane absorption with other bands that serve as a background reference. From recent literature in the field, we will be using two methods that create these methane enhancement products – we will use these both as baseline methods, and also as inputs to the later proposed machine learning models. These can be seen as classically extracted features.

We have included two methods into our analysis, namely the band ratio method proposed by^6^, which we will denote as “Varon ratio”, and the multi-linear regression (MLR) method proposed by^8^, which we will denote as the “Sanchez ratio”. In practice, while these methods were tested with different multispectral satellite data, either using the two SWIR bands of Sentinel-2 or the eight SWIR bands of WorldView-3, they remain sensor agnostic.

The work of^6^ proposed several methane enhancement methods, we use the mono-temporal variant that looks at the ratio between a signal band *S* and a background band *B*: $VaronRatio(S, B) = (c*S - B) / B$. The parameter *c* is used to scale one of the bands into the range of the other band, and can be obtained as a least square fit, or as a simplified formula $c = sum(B') / sum(S')$, where $S'$ and $B'$ corresponds to the signal and background bands with removal of outliers.

The method of^8^ instead uses multiple linear regression (MLR) to estimate the background information in the signal band from a combination of other bands. The estimated band $S_{MLR}$ is then compared with the signal band *S*: $SanchezRatio (S) = VaronRatio(S, S_{MLR})$. We note that the MLR estimation of each tile is not fitted on the whole training set, instead it uses only a single tile. The original paper uses the WorldView-3 bands, namely the B7 or the B8 as the signal bands and bands B1-B6 as background bands.

We will use the $\leftrightarrow$ symbol to refer to the Varon ratio, with first parameter being the signal band and the second parameter the background band. We explore these three variants: (1) First variant, denoted as “Varon”, uses the following ratios: B7$\leftrightarrow$B5, B8$\leftrightarrow$B5, and finally B7$\leftrightarrow$B6. (2) Second variant, denoted as “Sanchez”, uses the B1-B2 and B4-B6 as background bands to compute the MLR products: B7$\leftrightarrow$B7$_{MLR}$, B8$\leftrightarrow$B8$_{MLR}$, and the SWIR band B1. (3) Finally, the third variant, denoted as “Varon+Sanchez”, is a combination of the two previous methods – using first two Varon ratios with the first Sanchez ratio.

As the baseline method we use the Sanchez ratio computed for B8$\leftrightarrow$B8$_{MLR}$ thresholded by the experimentally found value of 0.05 and post-processing the binary output with the opening morphological operation. We have tested other ratio products as the baseline method, but the results were almost the same for all variants – the thresholded detections are very noisy regardless of the used ratio.

### Feature extraction from hyperspectral data

Hyperspectral data has very narrow wavelength windows at high spectral resolution, as can be seen on Fig. 2, which is crucial for methane detection. In such cases, it is easier to contrast bands inside and outside of the typical methane absorption to enhance the visibility of the plume inside the image. However, in practice, this approach would still result in a relatively large amount of noise in the extracted features, which is why the typical state of the art methods in this domain use matched filter approaches.

We build on top of the matched filter approach of mag1c^10^. A vanilla matched filter method measures, for every hyperspectral pixel, the similarity between the pixel value minus the average surface reflectance against the methane absorption spectrum (black line in Fig. 2). The proposal of^10^ improves the method by adding sparsity regularization and an albedo correction to the target spectrum to match. Although this method significantly reduces the amount of false positives, the retrieved image has still a high amount of noise; we found that this happens especially in urban areas (rooftops), water bodies, and human made infrastructures (photovoltaic panels, roads, etc.). As a baseline method we use the mag1c filter with the threshold of 500 ppm$\times$m and an opening morphological filter to remove the speckle noise.

### Machine learning models

**Figure 5:**
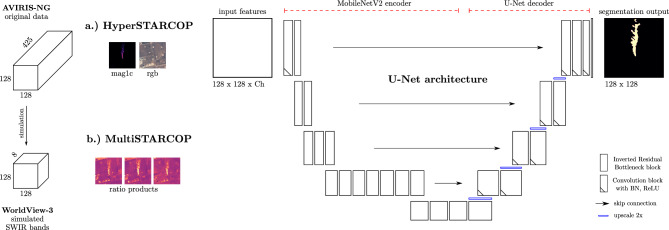
The proposed HyperSTARCOP and MultiSTARCOP machine learning models based on the U-Net architecture with MobileNetV2 as its encoder network. We note that this architecture is quite lightweight and it has only 6.6M parameters.

In this work, we propose two machine learning model variants, working with the multispectral and hyperspectral data – these two models however share the same architecture design illustrated in Fig. 5, except for using a different number of input channels.

We have chosen to use the U-Net architecture^16^ with MobileNet-v2 encoder^39^. Our HyperSTARCOP model is trained from scratch, while for the MultiSTARCOP variant, we use the encoder network pre-trained on the ImageNet dataset. This limits our choice of input bands to 3 to mimic the RGB bands commonly used in computer vision tasks, however, experimentally this led to better results with multispectral data and the MultiSTARCOP model. We use min-max normalisation for the ratio products and selected bands, using the statistics from the training dataset.

The **MultiSTARCOP model** uses ratio products computed from the WorldView-3 data. The challenge for this model remains in learning which part of the image contains a plume, and which contains the background information. We note that the ratio methods often highlight other structures present in the image with even stronger signal than that of the methane plume – as can be seen on Fig. 1 with highlighted building outline. In these cases, the signal of the methane is similar to other signatures present in the data. Our model has to learn to differentiate between the shapes and the strength of the signal corresponding to methane plumes and the other background classes. We also note that the strengths of different plumes vary quite significantly and, as such, the model needs to learn how to detect both weak and strong methane plume signatures. In initial exploratory experiments, we tried to train models on separate subsets of the data (such as data containing only strong plumes), but we saw a decrease in performance - we hypothesise that having a dataset of diverse plume shapes and sizes is beneficial.

The **HyperSTARCOP model** instead aims to improve upon the limitation of the current state of the art method of mag1c, namely in reducing its false positive rate. Our model uses the mag1c product with a selection of other bands from the hyperspectral sensor as input features. The underlying assumption is that a machine learning model should be able to learn which of the methane plumes outlined by the mag1c method are true plumes and which are just false detections. This information can be obtained either from the shape of the plume data, where the spatial information seen by 2D convolutional layers should outperform the per pixel baseline. We have tested two versions of this model, one relying only on the mag1c data as the input, and another using the mag1c product with addition of the RGB bands from the AVIRIS data (bands with central wavelengths 640 nm, 550 nm and 460 nm). The assumption is that if a human expert can distinguish between a falsely detected signal from a roof of a house and a real plume, then our model can learn the same.

## Experimental setup

The dataset we use for training the multispectral and the hyperspectral models contains the same samples, with just different views of the data – the original hyperspectral bands, or the simulated multispectral data corresponding to the bands of the WorldView-3 satellite. Correspondingly, we have taken similar approaches when training these two models, but in some instances, we used different hyperparameters.

For all training runs we use the Adam optimiser with learning rate of 0.001, keeping other parameters to their default values. In addition, we use a scheduler that reduces the learning rate on plateau by multiplying it with a factor of 0.5, with the patience parameter set to 4. Therefore, the training rate reduces only after 4 epochs without improvement. In total, we train for 15 epochs. The exact values for these hyper-parameters have been established experimentally. For development (training and validation), we use a n1-highmem-8 instance on Google Cloud Platform with one NVIDIA Tesla V100 GPU, one full training and validation run takes between 6 to 8 hours (depending on the used configuration and the number of input products).

The training dataset is heavily unbalanced in terms of the number of pixels corresponding to the plumes in contrast to the pixels corresponding to the background class. As such, we need to employ rebalancing measures. For both models, we oversample the instances from the minor class with the function provided by the PyTorch library, the WeightedRandomSampler. We take additional measures, but the approach differs for the multispectral and the hyperspectral scenario.

For training the MultiSTARCOP model we use weighted binary cross-entropy loss with the plume pixels weighted by the value of 15. For the HyperSTARCOP model we instead introduce a novel training loss for the context of the task of methane plume detection with hyperspectral data. We weight the loss by the mag1c product – this means that pixels with larger concentration values in the mag1c product will contribute more to the computed loss. We multiply the non-weighted binary cross-entropy loss computed over the whole tile with the mag1c product. This approach is similar to the one used in the original U-Net paper^16^, where a loss weight mask was used to prioritize pixels between individual segmented detections.

### Metrics

To evaluate our models, we first explore the raw outputs of our segmentation models. Secondly, we use a simple rule to convert these segmentation maps into classification decisions per each tile, to label them with the binary class of either having or not having any methane plume.

The segmentation results are described with the area under the precision-recall curve (AUPRC) score, which is independent to the used threshold, and works well in unbalanced scenarios. Each pixel of the segmentation map is then thresholded with the value of 0.5 to produce a binary map – which is then used to compute the F1, precision and recall statistics. For better insight into the performance of the model, we report these scores separately for strong plumes (with emission rate larger than 1000 kg/h) and for weak plume events. Each tile in the evaluation dataset is finally marked as containing a plume if the prediction has more than 10 active pixels. We study the false positive rate (FPR) on the subset of the evaluation dataset that does not contain any plumes. Additionally, we report the percentage of captured plumes stratified into several plume size categories. For a tile in the evaluation dataset which was predicted as containing a plume, we consider this plume to be captured if the thresholded prediction has at least 1 pixel overlap with the ground truth annotation.

### Experiments with generalization ability of our hyperspectral models

In the final experiment, we measure the capabilities of our model to serve as a zero-shot detector of methane leaks on data from other hyperspectral sensor. More concretely, we use the trained HyperSTARCOP models with inputs from the EMIT sensor. Given the knowledge of the wavelength ranges of each band in this new sensor, we can compute the required mag1c product. Furthermore, we re-normalise the RGB bands using the statistics from the AVIRIS training dataset, moving the data into the ranges expected by the models. While the deployment of machine learning models trained on standard computer vision datasets has been tested in-the-wild with other camera instruments (typically also RGB), the scenario with hyperspectral sensors is more complex as the exact number and location of bands, their specific noise profile and the ground spatial resolution differ. Despite these differences, we are able to compute similar input products, and reuse the pre-trained model in a zero-shot manner. We note that we do not alter the ground resolution of the data from EMIT (60 m). Given the diversity of the plumes present in our dataset, and namely their distribution across different sizes, we expect the model had to learn to be scale agnostic.

## Results

We present the results from: (1) training the MultiSTARCOP model on the simulated WorldView-3 data, (2) training the HyperSTARCOP model with the hyperspectral data from the AVIRIS sensor, and finally (3) evaluating the HyperSTARCOP model for zero-shot detection of methane leaks in the hyperspectral data from the EMIT sensor. The simulation of the multispectral views of the data from the original hyperspectral data is described in more detail in Section “AVIRIS machine learning ready dataset”. The metrics we use to analyze the performance of our models are described in Section “Metrics”.

We reiterate that we use annotation from real world plume leak events, and that a comparison between the different models is possible given the shared origin of the data. To illustrate this we show a single plume event on Fig. 1 evaluated with our proposed models in comparison with the appropriate baselines for the multispectral and the hyperspectral scenario. We note that the predictions of the baseline methods produce more false positive detections (shown as blue pixels), than any of our proposed models. When comparing the prediction between the two modalities of our proposed models, the MultiSTARCOP model is generally able to only detect the area of the plume with the higher gas concentration, while it misses the extended plume tail. On the other hand, the HyperSTARCOP model is capable of detecting the entire plume, including the areas of lower concentration in the plume tail.

**Figure 6:**
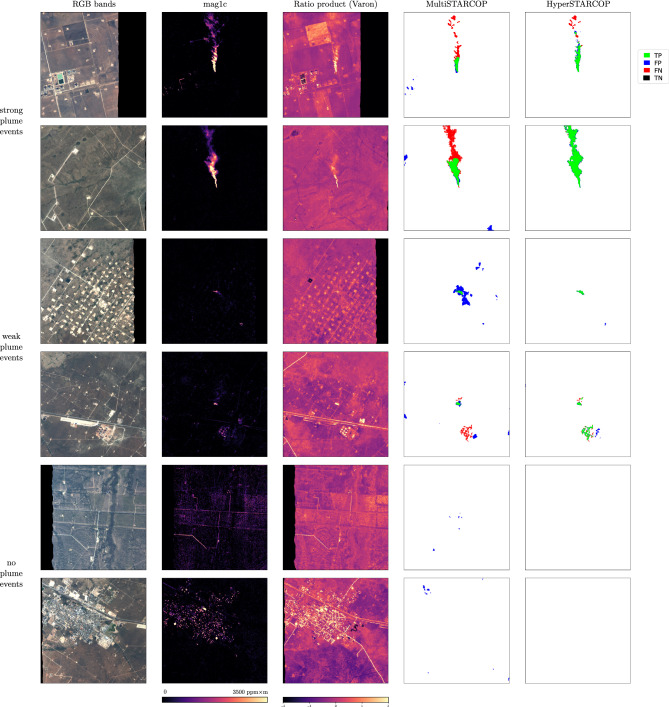
Showing the qualitative results of our models evaluated on strong plume samples (in the first two rows), on weak plume samples (in the second two rows) and finally on samples that are known as confounders (in the last two rows). Left to right columns in each sample shows: the RGB bands of the AVIRIS-NG data, the normalised mag1c product computed from the hyperspectral data, the normalised Varon ratios between bands B7 and B5 from the multispectral view of the data, prediction of the MultiSTARCOP model and the HyperSTARCOP model in comparison with the ground truth label. In the first three columns we show the areas outside of the sensor swath (no-data areas) with black background.

In Fig. 6, we show qualitative results of our models on diverse tiles from the evaluation dataset.

### Performance on multispectral data

We have trained the MultiSTARCOP model with the simulated WorldView-3 view of the data with three different enhancement product combinations. In Table 1, we show the segmentation and the classification scores of these three model variants. All of the proposed variants outperform the baseline approach in any of the used metrics. From the explored three variants, the model that uses the “Varon+Sanchez ratios” achieves the best performance in terms of the AUPRC and the F1 metrics. However, we note, that these results are within the range of standard deviation with the model that used the “Varon ratios”, which on the other hand receives the best (lowest) false positive rate by tile. We also see that the performance of all models drops rapidly with smaller plume events, which confirms the assumption that the detection of small methane plumes is a challenging task.

**Table 1:** Results of the multispectral models on the test set, we show results of our proposed model in comparison with the existing baseline. We show the average results of 5 training runs of our models.

	F1 (strong) $\uparrow$	F1 (weak) $\uparrow$	FPR by tile $\downarrow$	AUPRC $\uparrow$
Baseline, ratio + morpho.	7.44	0.5	100.0	N/A
Our (Varon)	30.72 ± 2.87	10.35 ± 1.52	**87.89 ± 4.67**	11.92 ± 1.35
Our (Sanchez)	26.59 ± 3.13	9.32 ± 1.05	94.4 ± 1.30	9.96 ± 1.43
Our (Varon+Sanchez)	**31.89 ± 2.44**	**11.04 ± 0.75**	90.51 ± 4.23	**13.04 ± 1.96**

Best performing values are in bold.

### Performance on hyperspectral data

In Table 2 we report the results of training our HyperSTARCOP model on the AVIRIS data using two different input configurations. We see that the proposed HyperSTARCOP model variants both outperform the baseline approach. We see an increase in the F1 score across both strong (emission larger than 1000 kg/h) and weak plume events, while at the same time achieving a decrease in the false positive rate. This means that the proposed method produces better semantic segmentation of the methane plumes, while also being less sensitive to noise.

**Table 2:** Results of the hyperspectral models on the entire test set, we show results of our proposed model in comparison with the existing baseline. We show the average of training 5 runs of our models.

	F1 (strong) $\uparrow$	F1 (weak) $\uparrow$	FPR by tile $\downarrow$	AUPRC $\uparrow$
Baseline, mag1c + morpho.	67.45	39.95	75.43	N/A
HyperSTARCOP, only mag1c	74.15 ± 6.10	**47.57 ± 4.17**	52.11 ± 10.98	49.41 ± 5.49
HyperSTARCOP, mag1c + rgb	**81.96 ± 3.71**	43.42 ± 5.72	**43.66 ± 7.36**	**51.99 ± 2.76**

Best performing values are in bold.

**Figure 7:**
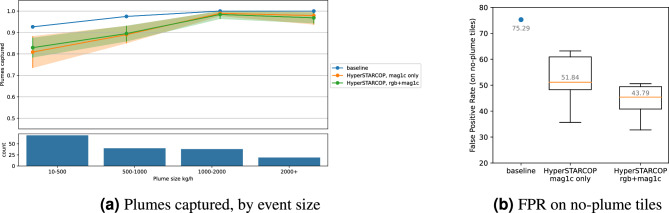
Results on the test set using fine grade distinction between plume sizes (average of 5 runs of our models).

Furthermore, we explore a more fine-grained evaluation of the proposed models using the per tile classification scores. In Fig. 7a, we explore the percentage of captured plumes stratified by different plume emission sizes, showing the natural trend that stronger plumes are easier to detect. Both of the proposed model variants achieve similar performance. In comparison the mag1c baseline captures more plume tiles – as it predicts more tiles as containing plumes in general. In Fig. 7b, we show that this leads to larger false positive rate on no-plume tiles – the baseline method gets the FPR of 75.29. Both of the proposed models are able to significantly reduce the FPR, with the “mag1c+rgb” model outperforming the “mag1c only” variant. We also note that the FPR on no-plume tiles reported in Fig. 7b is similar to the score of FPR in Table 2, which is evaluated on all tiles (including the ones with plumes).

In summary, on no-plume tiles, the HyperSTARCOP “mag1c+rgb” variant achieves the FPR score of 43.79, reducing the FPR by over 41.83% in contrast to the baseline. Furthermore, we see better performance in the segmentation statistics, namely increase of the F1 score for strong (by 21.51%) and weak (by 8.68%) plume events in comparison with the baseline. When using a simple rule to convert these segmentation predictions into per-tile classification, we see a mostly maintained performance, with a small drop in the detection capabilities of the weak methane plume events. We note that this is consistent with the fact that the model was trained on the task of semantic segmentation, and that there are likely more complex methods available for generating per tile classifications.

### Zero-shot generalisation on EMIT

**Figure 8:**
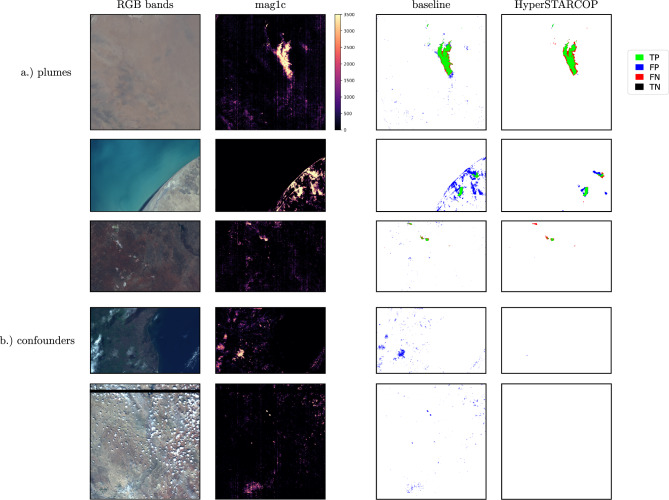
Results on example plume and confounder samples from the EMIT dataset, showing the baseline method and our HyperSTARCOP model using mag1c+rgb as inputs.

We use the HyperSTARCOP models trained on our dataset from the AVIRIS sensor in a zero-shot manner with new data from the EMIT sensor. Table 3 shows that the performance of our proposed model outperforms the baseline approach in F1 score by up to 40.28%. Furthermore the “mag1c+rgb” variant gets better performance in all metrics in comparison with the “mag1c only” version. We also note that the standard deviation of these results is quite high, which is consistent with the fact that the models have to generalise on unseen data and cope with the associated spectral and spatial biases.

**Table 3:** Results of the hyperspectral models on EMIT. We show the average of training 5 runs of our models.

	F1 $\uparrow$	Precision $\uparrow$	Recall $\uparrow$	AUPRC $\uparrow$
Baseline on EMIT	35.25	24.08	**65.8**	N/A
HyperSTARCOP, only mag1c	42.54 ± 5.22	68.93 ± 4.72	31.09 ± 5.43	46.61 ± 4.61
HyperSTARCOP, mag1c + rgb	**49.45 ± 10.79**	**73.17 ± 7.40**	38.78 ± 12.48	**53.29 ± 8.08**

Best performing values are in bold.

On Fig. 8 we selected few plume events and areas with typical confounders for the mag1c product (urban areas). We see that our HyperSTARCOP model is capable of detecting methane plume events, while also being able to reject the detection falsely highlighted in the mag1c product.

We note that these are highly desired properties that have been transferred from the AVIRIS dataset onto evaluated data from another sensor. A typical next step would be to further use our trained models and to finetune them on labelled EMIT datasets. However, the number of labelled examples present in the EMIT dataset so far is much lower than the number of events in the AVIRIS dataset. To summarize, we see that our model has learned useful representations that allow for zero-shot generalisation on data from other sensors. Our models achieve better qualitative and quantitative results, namely they improve the F1 score of the baseline approach by 40.28%.

## Conclusion

In this work, we explore semantic segmentation of methane plumes in the hyperspectral and multispectral data with machine learning models. We publicly release a large scale and high resolution dataset of hyperspectral images from the AVIRIS-NG sensor. We have refined the existing labels^15^, improving the annotation which is required for training machine learning models. We provide the raw hyperspectral data alongside with simulated multispectral views of the same data, allowing for direct comparison between the two modalities of data. We hope that this dataset will promote research in the areas of methane detection and processing hyperspectral data with machine learning models.

We propose and evaluate models based on the small and efficient U-Net architecture with MobileNetV2 encoder with several different configurations. The resulting model architectures are lightweight, with only 6.6M parameters. Our experiments with simulated WorldView-3 data, showcases the difficulties of detecting methane plumes in data from multispectral instruments. On strong plume events, our MultiSTARCOP models get the average F1 score of 31.89 outperforming the multispectral baseline which has F1 score of only 7.44. Our proposed HyperSTARCOP model outperforms the state-of-the-art baseline approach of mag1c^10^ obtaining better performance in methane plume segmentation, namely increasing the F1 score for strong events by 21.51% and weak events by 8.68%. Importantly, our model also addresses the known limitation of matched filter methods, which produces many false positive detections. We reduce the false positive rate per tile by over 41.83% in contrast to the baseline, at the cost of small drop in the number of captured plumes.

Finally, we show that our HyperSTARCOP model can be used for zero-shot generalisation on data from another hyperspectral sensor. Without fine-tuning the model, we obtain a superior score to the mag1c approach on data from the new EMIT sensor, on previously unseen locations. On a small, annotated evaluation set, we improve the F1 score on average by 40.28% over the baseline method. The initial results with the EMIT dataset provide interesting avenues for follow-up research in zero or few-shot learning with hyperspectral data. Furthermore, with the publicly released data, we provide a benchmark dataset to compare machine learning models for hyperspectral data processing, which has been highlighted as crucial by numerous recent overview studies and works^22,24–26^.

As potential future research directions we see developing specific architectures for processing hyperspectral data, for which the very recent pre-print of^40^ is a promising direction. Alternatively, we would like to point towards exploration of general, sensor-agnostic systems, that would be able to detect signals of arbitrary gas signatures from hyperspectral data. Another avenue would be in pursuing development of lightweight models for deployment on-board a satellite. This will allow intelligent decision making in Space for near-real time alerting. This will require evaluating the speed of the trained models in a constrained environment with data available directly on the device, similarly as was done in^41,42^ in the cases of disaster event and flood detection models.

## Data Availability

We are releasing the full annotated training and test STARCOP datasets on Zenodo https://doi.org/10.5281/zenodo.7863343^38^, the code and the pre-trained models alongside this paper at https://github.com/spaceml-org/STARCOP. We further note that all figures of this paper have been produced with open source python libraries matplotlib, rasterio and folium. Original AVIRIS-NG imagery was gathered from the AVIRIS-NG data portal. EMIT imagery^43^ was downloaded from the NASA Earth data portal.

## Duplicate publication statement

An initial and shorter version of this work was previously presented at the ESA-ECMWF workshop of 2022 as an oral presentation (publicly available here). This paper has been significantly updated since the presentation, with the evaluation on the EMIT sensor.
